# Comprehensive Investigation of Cu^2+^ Adsorption from Wastewater Using Olive-Waste-Derived Adsorbents: Experimental and Molecular Insights

**DOI:** 10.3390/ijms25021028

**Published:** 2024-01-14

**Authors:** Noureddine Elboughdiri, Hana Ferkous, Karima Rouibah, Abir Boublia, Amel Delimi, Krishna Kumar Yadav, Alessandro Erto, Djamel Ghernaout, Alsamani A. M. Salih, Mhamed Benaissa, Yacine Benguerba

**Affiliations:** 1 Chemical Engineering Department, College of Engineering, University of Ha’il, P.O. Box 2440, Ha’il 81441, Saudi Arabia; djamel_andalus@yahoo.fr (D.G.); samani15@hotmail.com (A.A.M.S.); m.benaissa@uoh.edu.sa (M.B.); benguerbayacine@yahoo.fr (Y.B.); 2 Laboratoire de Génie Mécanique et Matériaux, Faculté de Technologie, Université de Skikda, Skikda 21000, Algeria; h.ferkous@univ-skikda.dz (H.F.); a_delimi03@yahoo.fr (A.D.); 3 Laboratory of Materials-Elaborations-Properties-Applications (LMEPA), University of MSBY Jijel, PB98 Ouled Aissa, Jijel 18000, Algeria; karima.rouibah@univ-jijel.dz; 4 Laboratoire de Physico-Chimie des Hauts Polymères (LPCHP), Département de Génie des Procédés, Faculté de Technologie, Université Ferhat ABBAS Sétif-1, Sétif 19000, Algeria; abir.boublia@univ-setif.dz; 5 Faculty of Science and Technology, Madhyanchal Professional University, Ratibad, Bhopal 462044, India; envirokrishna@gmail.com; 6 Environmental and Atmospheric Sciences Research Group, Scientific Research Center, Al-Ayen University, Thi-Qar, Nasiriyah 64001, Iraq; 7 Dipartimento di Ingegneria Chimica, dei Materiali e della Produzione Industriale, Università di Napoli Federico II, 80125 Napoli, Italy; aleserto@unina.it; 8 Laboratoire de Biopharmacie et Pharmacotechnie (LBPT), Université Ferhat ABBAS Sétif-1, Sétif 19000, Algeria

**Keywords:** copper ion adsorption, sodium alginate-modified olive waste adsorbent, density functional theory (DFT)

## Abstract

This study investigates the efficacy of adsorbents from locally sourced olive waste—encompassing olive skins, leaves, and pits, recovered from the initial centrifugation of olives (OWP)—and a composite with sodium alginate (OWPSA) for the removal of Cu^2+^ ions from synthetic wastewater. Experimental analyses conducted at room temperature, with an initial Cu^2+^ concentration of 50 mg/L and a solid/liquid ratio of 1 g/L, showed that the removal efficiencies were approximately 79.54% and 94.54% for OWP and OWPSA, respectively, highlighting the positive impact of alginate on adsorption capacity. Utilizing statistical physics isotherm models, particularly the single-layer model coupled to real gas (SLMRG), allowed us to robustly fit the experimental data, providing insights into the adsorption mechanisms. Thermodynamic parameters affirmed the spontaneity and endothermic nature of the processes. Adsorption kinetics were interpreted effectively using the pseudo-second-order (PSO) model. Molecular modeling investigations, including the conductor-like screening model for real solvents (COSMO-RS), density functional theory (DFT), and atom-in-molecule (AIM) analysis, unveiled intricate molecular interactions among the adsorbent components—cellulose, hemicellulose, lignin, and alginate—and the pollutant Cu^2+^, confirming their physically interactive nature. These findings emphasize the synergistic application of experimental and theoretical approaches, providing a comprehensive understanding of copper adsorption dynamics at the molecular level. This methodology holds promise for unraveling intricate processes across various adsorbent materials in wastewater treatment applications.

## 1. Introduction

In recent years, the global increase in population and widespread industrial activities have resulted in the release of untreated wastewater containing toxic metals, presenting a significant environmental challenge. The presence of non-degradable hazardous substances in the environment has led to various issues due to their toxicity. Therefore, a primary objective in environmental science research is to develop effective methods and technologies for treating such toxic effluents [1]. Water contamination originates from human communities, industries, and agriculture, with 80% of globally produced wastewater being discharged untreated into aquatic bodies. The industrial sector, in particular, is a major contributor, releasing millions of tonnes of heavy metals annually [2]. Water pollution caused by heavy metals is a global concern due to their harmful effects on humans and other organisms and their tendency to bioaccumulate, making traditional methods ineffective [3].

In the present-day understanding, the term "heavy metal" encompasses metallic elements and metalloids acknowledged for their inherent toxicity to both human health and the environment [4,5]. Industries engaged in galvanic processes, paint manufacturing, photography, surface treatment, and electronic circuitry extensively utilize heavy metals, many of which are carcinogenic, thereby posing substantial risks to human health and the aquatic ecosystem [6]. Due to widespread sources of pollution, the World Health Organization (WHO) has been compelled to regulate heavy metals and their industrial discharge into the environment [7]. Among the most hazardous heavy metals are lead (Pb), mercury (Hg), arsenic (As), hexavalent chromium (Cr^6+^), and cadmium (Cd). However, some metallic trace elements—essential oligo-elements for living beings—include copper (Cu), zinc (Zn), iron (Fe), manganese (Mn), trivalent chromium (Cr^3+^), and selenium (Se). It is noteworthy that, despite their essential nature, these elements can become toxic and harmful to ecosystems above a certain concentration. Copper, widely employed in various applications, poses a significant risk of contamination in water resources [8]. This risk underscores the importance of our research focus on copper removal from wastewater. Specifically, our emphasis on reducing the concentration of copper in industrial effluents to permissible limits aligns with the critical need to safeguard water quality and environmental health [9].

Various techniques, such as precipitation, ion exchange, electrolysis, membrane processes and coagulation–flocculation have been used to remove toxic heavy metals from wastewater. However, these traditional methods have drawbacks, including high chemical and energy demands, the generation of potentially dangerous sludge, lower removal efficiency at low metal concentrations, and high operational costs at a large scale [10]. Adsorption has been successful in the removal of trace metal elements due to its ease of use, high capacity to eliminate these pollutants at low concentrations, and its low cost compared to other treatment methods [11]. While activated carbons have been extensively used for heavy metal removal, they are often expensive and require additional modifications [12]. Recent efforts have explored alternative biomaterials, including tree fern, cottonseed hulls, sawdust, banana pips, and peat, for cost-effective and sustainable wastewater treatment [13,14,15,16,17,18].

Optimizing adsorption systems is crucial for enhancing technology [19,20]. Molecular-based in silico approaches, such as density functional theory (DFT), the conductor-like screening model for real solutions (COSMO-RS) [21,22], and other advanced modeling techniques, have proven valuable in providing insights into adsorption mechanisms at a molecular level [22]. Additionally, the atom-in-molecule (AIM) theory, introduced by Bader, has found extensive applications in the elucidation of diverse interactions within various molecular systems, and involves the analysis of bonding interactions through real-space functions, particularly focusing on electron density at bond-critical points (BCPs) [23,24,25]. These sophisticated models offer a powerful means to predict and understand the behavior of adsorbents and adsorbates. However, to bridge the gap between theory and experiment, further work is needed to integrate and correlate experimental observations with these theoretical modeling techniques. In doing so, a more comprehensive understanding of the adsorption process, particularly at the molecular level, can be achieved [26,27,28].

In the present study, we explored the efficacy of utilizing raw olive waste powder (OWP) derived from waste olive residue and sodium alginate bio-composite beads (OWPSA) as environmentally friendly and cost-effective bio-adsorbents for the removal of copper ions (Cu^2+^) from aqueous solutions. The incorporation of sodium alginate in the form of biosorbent beads presents several advantages over its raw counterpart. The encapsulation of waste using alginate serves a dual purpose: to prevent particle agglomeration in solutions and to enhance copper adsorption capacity. Alginate, chosen for its known high affinity to metals, acts as a biopolymer that improves the overall performance of an adsorbent. It is worth mentioning that the standalone use of alginate in water treatment is constrained by its tendency to swell in water and its mechanical weaknesses. The findings of this study reveal that olive waste possesses favorable adsorbent properties, and these properties are further enhanced through encapsulation with alginate. A rigorous characterization of the adsorbents and systematic adsorption experiments was conducted to unravel their thermodynamic and kinetic characteristics. Utilizing various models from the realm of physical statistics, we meticulously analyzed the experimental data to gain valuable insights into the adsorption process. Moreover, we employed molecular modeling analyses, including physical statistics, density functional theory (DFT) computations, a COSMO-RS study, and an AIM analysis. These sophisticated techniques were instrumental in deepening our understanding of the intricate interactions between the adsorbents and Cu^2+^ ions at the molecular level. This comprehensive approach not only validates the potential of the proposed bio-adsorbents, but also provides a nuanced understanding of their performance for future advancements in heavy-metal-removal applications.

## 2. Results and Discussion

### 2.1. Adsorbent Characterization

**Figure 1:**
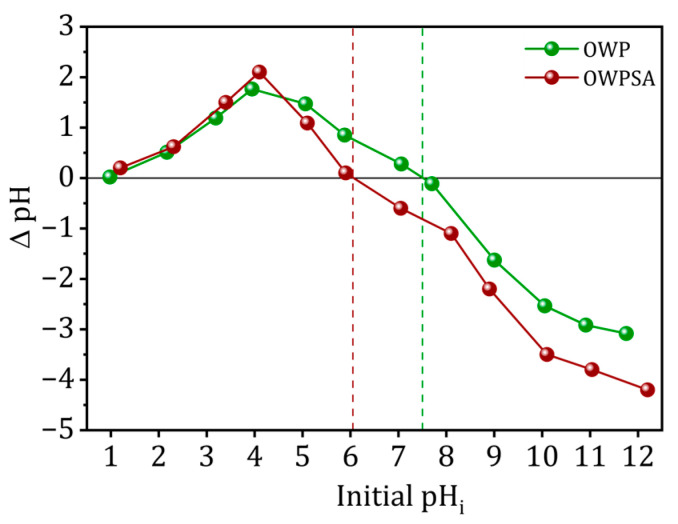
pH_pzc_ of OWP and OWPSA adsorbents.

The pH_pzc_ values for the adsorbents under investigation are shown in Figure 1. A pH_pzc_ of 7.6 was recorded for olive waste powder (OWP), indicating that its outermost layer is either neutral or slightly alkaline. When olive waste powder was combined with sodium alginate, the resultant material was named olive waste powder–sodium alginate (OWPSA) and had a pH_pzc_ of 6.1, suggesting a mildly acidic surface. The influence of alginate encapsulation on the adsorbent’s surface characteristics is shown by a shift from neutral to slightly acidic pH_pzc_ values.

**Figure 2:**
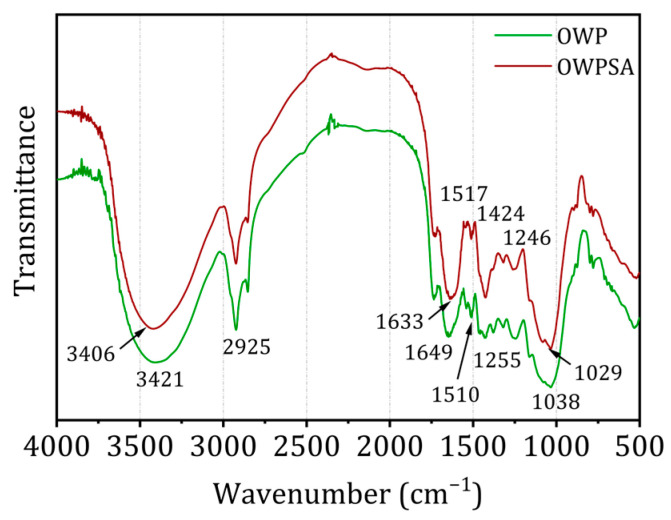
OWP and OWPSA FTIR spectra.

Using infrared spectra generated by Fourier transform infrared (FTIR) spectroscopy, it is possible to discern the functional groups present on the surface of the adsorbent. Figure 2 displays the Fourier transform infrared (FTIR) spectra of OWP and OWP–sodium alginate adsorbents (OWPSA).

FTIR spectra may exhibit absorption bands that are indicative of lignocellulosic chemicals. A notable absorption band is produced at 3406 cm^−1^ (OWP) and 3421 cm^−1^ (OWPSA) correspond to the vibrations of elongation of hydrogen bonds in the hydroxyl group (OH). This collective comprises a multitude of functional groups, such as phenols, alcohols, carboxylic acids, and adsorbed water [29]. The visible band results from the elongation of the O-H bond in cellulose, hemicellulose, and lignin [30]. This information provides crucial insights into the chemical composition of the adsorbents and assists in identifying surface functional groups essential for adsorption interactions. Furthermore, it is essential to underscore the consistent shape of the curves in the FTIR spectra for both OWP and OWPSA to underscore the robustness and reliability of our observations. An absorption band is seen in the spectra of both adsorbents at around 2925 cm^−1^. The stretched peaks in the methyl and methylene structures probably correspond to the C-H bonds. Additional OWP bands may be seen at 1518–1425 and 1650 cm^−1^, corresponding to lignin’s C=C peaks of olefinic and aromatic compounds. Analogous vibrations may be seen in OWPSA at 1633 cm^−1^ and throughout the frequency range of 1510 to 1424 cm^−1^. The 1246 and 1255 cm^−1^ bands, which correspond to OWP and OWPSA, might indicate phenolic groups, ethers, or esters. Alcohol group vibrations at 1038 and 1029 cm^−1^ are ascribed to R-OH bonds [31,32].

### 2.2. Adsorption Study

**Figure 3:**
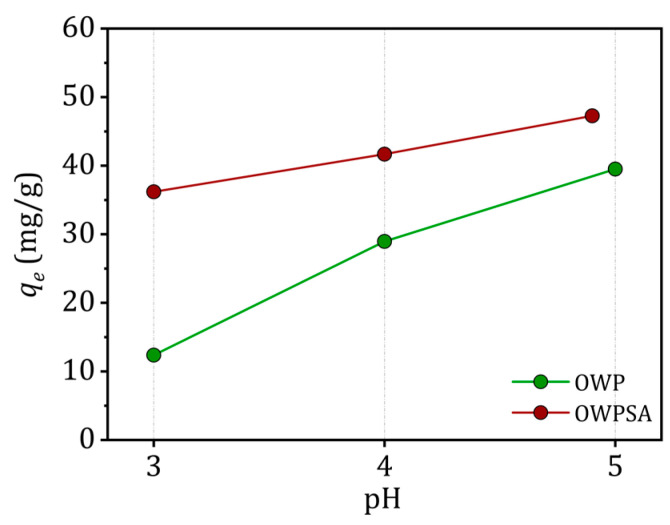
Effect of pH (conditions: *m* = 20 mg, *C*_0_ = 50 mg/L, *V* = 20 mL, *T* = 303 K).

The impact of solution pH on OWP’s and OWPSA’s adsorption capacity for Cu^2+^ ions is illustrated in Figure 3. The experimental findings reveal an enhancement in the adsorption of Cu^2+^ by both adsorbents as the pH of the solution increases. Notably, the adsorption capacity undergoes a substantial increase as the pH value rises from the lowest point considered (3). Cu^2+^ removal from the solution continues to improve beyond a pH of 5. However, a decline in the adsorption of Cu^2+^ is observed at low pH levels, attributed to repulsion between the adsorbent and cationic ions.

Importantly, as pH values surpass the pH_pzc_, a negatively charged surface is generated due to a decrease in H^+^ proton concentration, leading to electrical attraction between the adsorbents and Cu^2+^ ions. It is crucial to acknowledge the influence of precipitation in the Cu sorption process, particularly at pH > 5.5, where Cu tends to precipitate in the form of hydroxide [33].

**Figure 4:**
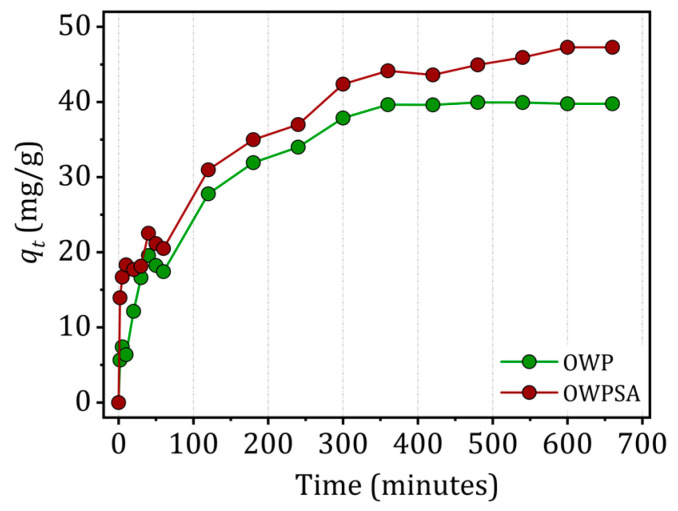
Effect of contact time (conditions: *m* = 20 mg, *C*_0_ = 50 mg/L, *V* = 20 mL, *T* = 303 K).

Adsorption kinetics is a major factor in selecting an appropriate system size. The time required to reach adsorption equilibrium might differ depending on the system under study and the operational parameters [29]. Figure 4 shows how the ratio of Cu^2+^ ions adsorbed by OWP to those adsorbed by OWPSA changes over time. Specifically, the quantity of adsorbed Cu^2+^ on OWP increases with time, reaching equilibrium at 360 min. The adsorption capacity at this point is 39.77 mg/g, with an efficiency of 79.54%.

Conversely, the adsorption of Cu^2+^ is a more sluggish process in the case of OWPSA, reaching equilibrium after about 600 min. Nevertheless, the ultimate adsorption capacity is greater, amounting to 47.2 mg/g, with a corresponding removal efficiency of 94.5%. This indicates a significant affinity between Cu^2+^ and the OWPSA support. Understanding the kinetics of Cu^2+^ adsorption is essential for optimizing operational parameters and ensuring the efficiency of the adsorption process in practical applications.

### 2.3. Adsorption Test Modelling

#### 2.3.1. Adsorption Kinetic Models

Two surface reaction models describe the kinetic experiments [34,35]: the PFO (pseudo-first order) and PSO models.

(1) $$\log(q_{e}-q_{t\;})=\log q_{e}-\frac{k_{1}}{2.303}\;t$$

(2) $$\frac{t}{q_{t}}=\frac{1}{k_{2}q_{e}^{2}}+\frac{1}{q_{e}}t$$

where *k*_1_ and *k*_2_ are, respectively, the pseudo-first and -second kinetic rates.

The intra-particle diffusion model was tested by using the equation described by Weber and Morris [35,36]:

(3) $$q_{t\;}=K_{id}t^{0.5}+C$$

where *q_t_* (mg/g) is the instantaneous adsorption capacity, *K_id_* is the intra-particle diffusion rate constant, and *C* is the thickness of the boundary layer.

**Figure 5:**
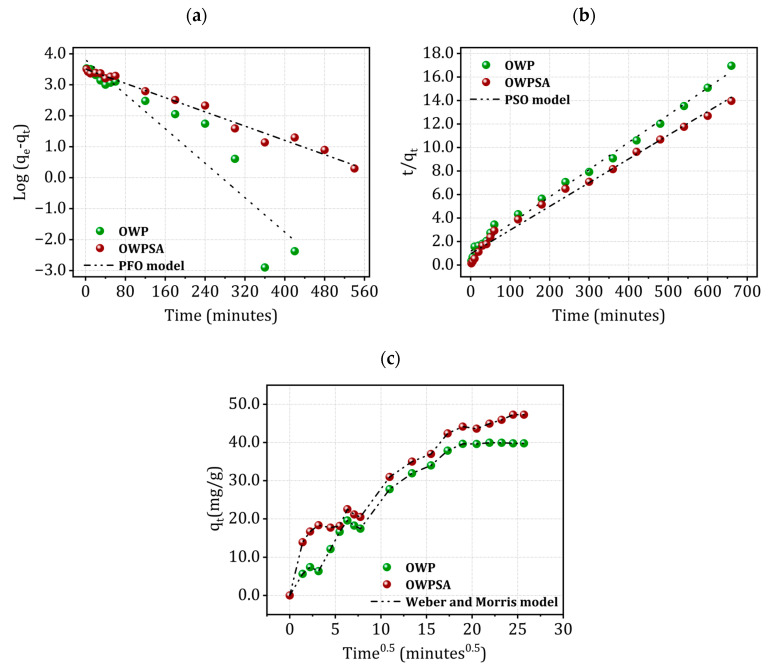
Kinetics of Cu^2+^ adsorption using (**a**) PFO, (**b**) PSO, and (**c**) Weber and Morris models.

Figure 5 displays the PFO, PSO, and intra-particle diffusion models for the kinetics of Cu^2+^ adsorption on OWP and OWPSA, respectively.

In our study, we utilized PFO, PSO, and Weber and Morris models to understand the kinetics of Cu^2+^ adsorption onto OWP and OWPSA. The obtained kinetic parameters, detailed in Table S1 of the Supplementary Material, were subjected to rigorous analysis to ascertain the most appropriate model for our system.

Upon thorough examination, the PSO model emerged as the superior choice, primarily driven by the coefficient of determination (R^2^). The PSO model exhibited a remarkable agreement between predicted and actual adsorption capacities, as visually depicted in Figure 5b. However, the application of the PSO model suggests that the main mechanism of adsorption is probably chemisorption, which may be rate-limiting step [37,38].

Meanwhile, as shown in Figure 5c, the curve *q_t_* = f (t^0.5^) is not linear and highlights the presence of three successive steps. The first one is attributed to adsorption on the external surface of the solid (external diffusion), the second is attributed to intra-particle diffusion (diffusion into the pores), and the last step corresponds to an equilibrium state. The intra-particle diffusion of Cu^2+^ is characterized by a straight line not passing through the origin, which means that if intra-particle diffusion is involved in the kinetic process, it does not constitute a step limiting adsorption [35,39].

#### 2.3.2. Adsorption Isotherm Models

Adsorption isotherms serve as crucial tools in defining the limits of the adsorption process, offering insights into the adsorption mechanism, surface properties, and the affinity level with the adsorbent. In examining the adsorption isotherm for Cu^2+^ onto OWP, OWPSA, and alginate, the experimental data were modeled using the single-layer model coupled to real gas (SLMRG). This model, derived from statistical physics, employs a grand canonical ensemble to study the adsorption process, which involves the transfer of particles between the free and adsorbed phases. It is assumed that any specified receptor site can be either occupied (*N*_i_ = 1) or unoccupied (*N*_i_ = 0) [40].

The theoretical expression for the SLMRG model is as follows:

(4) $$q_{e}=\frac{nN_{m}}{1+{\left(w\frac{1-bC_{e}}{C_{e}}e^{2a\beta \;C_{e}}\;e^{-\frac{bC_{e}}{1-bC_{e}}}\right)}^{n}}$$

n  indicates the number of adsorbed molecules at a given site, $N_{m}\left(\mathrm{mg}/g\right)$ denotes the density of receptor sites, $b\;\left(L/\mathrm{mg}\right)$ is the co-volume of the adsorbate, $a\;\left(J\;L/\mathrm{mol}\right)$ is cohesion pressure, β is a constant that can be written as ( $1/k_{B}T$ ), where $k_{B}$ is the Boltzmann’s constant, and w (J/mol) is the energetic parameter that is described as follows:

(5) $$w=Z_{gtr}e^{-\beta \varepsilon }=C_{s}e^{-\frac{E^{a}}{RT}}$$

**Figure 6:**
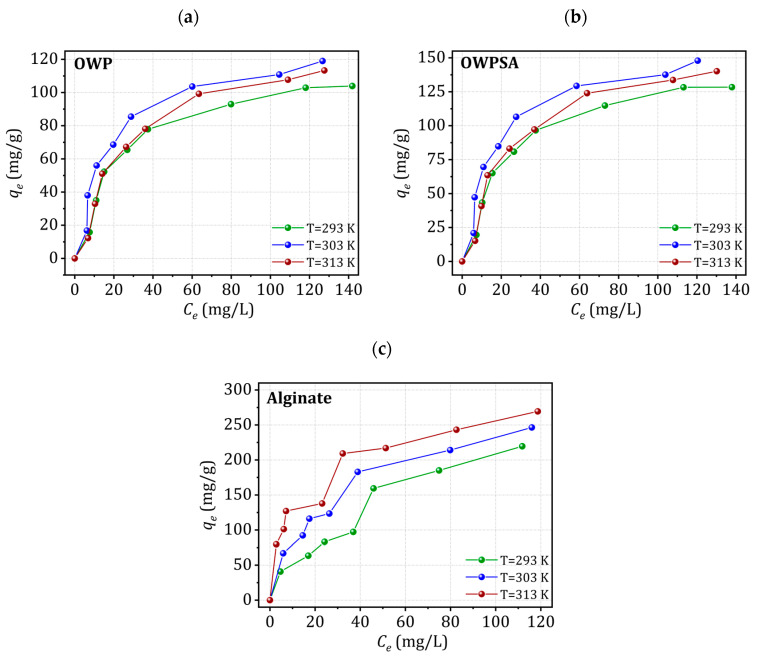
Copper adsorption isotherms for (**a**) OWP, (**b**) OWPSA, and (**c**) alginate.

Figure 6 presents the Cu^2+^ adsorption isotherms onto OWP, OWPSA, and alginate. Notably, all materials exhibit an isotherm characteristic of the Langmuir’s L group, indicating a specific adsorption behavior. As the initial concentration of copper in the solution varies, the amount adsorbed by OWP, OWPSA, and alginate exhibits a similar trend, steadily increasing until a saturation plateau is achieved. This plateau is indicative of the point at which all available adsorption sites on the surfaces of the OWP, OWPSA, and alginate are fully occupied by copper ions. Importantly, the plateau in the OWPSA isotherm is notably more pronounced than that for OWP or alginate, suggesting a higher adsorption capacity.

The enhanced performance of OWPSA compared to OWP or alginate is a crucial observation, pointing to the positive influence of incorporating sodium alginate. The synergy between OWP and sodium alginate contributes to a more efficient copper adsorption process, reaching saturation at a lower concentration. This improvement can be attributed to the combined effect of the individual strengths of OWP and sodium alginate, showcasing their complementary roles in enhancing adsorption capacity.

The isotherms not only emphasize the effectiveness of both materials at capturing copper ions from the solution, but also underscore the significance of their homogeneous adsorption behavior. The presence of sodium alginate further promotes ionic interactions, ensuring a uniform adsorption process. These findings highlight the potential of OWP and OWPSA in practical applications for wastewater treatment where efficient copper removal is paramount.

The adsorption isotherm modeling curves of Cu^2+^ onto OWP, OWPSA, and alginate at various temperatures are depicted in Figure S1 in the Supplementary Material. The SLMRG model exhibits excellent fitting between the experimental isotherm and theoretical data. This model provides a robust description of the experimental data and accounts for adsorbate–adsorbent interactions, significantly influencing adsorption competition and overall performance [40,41,42]. The SLMRG fitting parameters offer specific physical meanings, contributing to a deeper understanding of Cu^2+^ adsorption isotherms that can be utilized to analyze the experimental data of Cu^2+^ removal by OWP, OWPSA, and alginate. Table 1 presents the model results along with the fitting parameters. The SLMRG provides a robust description of the experimental data for OWP, OWPSA, and alginate, offering a reasonable interpretation of the temperature effect.

As shown in Table 1, the molecular cohesion pressure, a, represents the cohesive forces within the adsorbent materials, influencing their interactions with copper ions. The similarity in the orders of magnitude across OWP, OWPSA, and alginate indicates a uniformity in molecular interactions and adsorption behavior. This suggests that these adsorbents share comparable steric effects and mechanisms for capturing copper ions from the solution. Furthermore, the maximum adsorption capacity, a key parameter, is determined by the product of two sterically characteristic parameters: *n* and *Nm* [43]. This implies that the capacity to adsorb copper ions is influenced by the number of adsorbed molecules at *n* and *Nm*. The uniformity in these parameters across the different adsorbents further supports the consistent behavior observed in their Cu^2+^ adsorption isotherms.

In summary, the close resemblance in molecular cohesion pressure and other sterically characteristic parameters among OWP, OWPSA, and alginate underscores their similar adsorption mechanisms and capacities. These findings enhance our understanding of adsorption processes and contribute to predicting the performance of these adsorbents in removing copper ions from solution.

**Figure 7:**
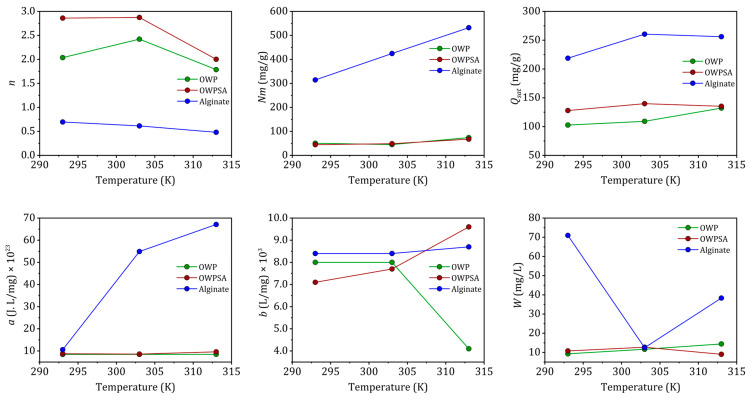
The variation in SLMRG parameters with temperature.

Figure 7 illustrates the change in the two steric parameters as a function of temperature. The *n* parameter, representing the cooperativity or competition of adsorption sites, is a crucial indicator in understanding the adsorption process. Typically ranging between 1 and 10, values closer to 1 suggest a more favorable and cooperative adsorption, while values greater than 1 indicate a competitive adsorption process. In our study, the *n* values for the Cu^2+^–OWP, Cu^2+^–OWPSA, and Cu^2+^–alginate systems at 293 K indicate a multi-interaction process, where the Cu^2+^ cation may interact with either half (1/2.037), a third (1/2.86), or one and a half (1/0.696) of the available adsorption sites. These variations in the *n* parameter highlight the diverse and complex nature of the adsorption mechanisms for different systems, providing valuable insights into the interactions between copper ions and the adsorbents under varying conditions [44]. Furthermore, as the temperature increases, *n* decreases for Cu^2+^–OWPSA and Cu^2+^–alginate, while for Cu^2+^–OWP, *n* rises, reaching a maximum at 303 K, and then decreases.

As described by the *Nm* values, the number of adsorption sites available to the adsorbate molecule increases proportionally to the increase in 1/*n* (number of adsorbing sites available for one adsorbate molecule). The endothermic nature of the adsorption process at elevated temperatures benefits the adsorption capacity, leading to a higher number of available adsorbing sites (1/*n*) for Cu^2+^ adsorption.

At higher temperatures, the adsorbed amount and molecular cohesion pressure “*a*” increase, indicating greater lateral interactions between adsorbate molecules. Although the exothermic increase hinders the endothermic process of adsorption in lateral contacts (a reduction in *n* at higher temperatures), the increase in available sites with increasing temperature counterbalances this loss. This effect is more pronounced when adsorbate interactions are stronger at higher temperatures. The co-volume “*b*” also increases as the intermolecular distance between adsorbates increases, resulting in higher adsorbed quantities.

Cu^2+^–alginate demonstrates notably higher values of ‘*b*’ across various temperatures, signifying its superior adsorption capacity in comparison to the other two systems, Cu^2+^–OWP and Cu^2+^–OWPSA. The energetic parameter ‘*w*’ can be determined using Equation (5). In the cases of OWP and OWPSA, ‘*w*’ exhibits an increase with temperature, while for alginate, it experiences a decrease. This observed decrease in ‘*w*’ for alginate suggests a reinforcement of the interaction between Cu^2+^ ions and the alginate adsorbent, indicating an augmentation of the adsorption energy modulus. The introduction of OWP to alginate further amplifies the adsorption of Cu^2+^ ions onto alginate, as evidenced by the decrease in ‘*w*’. This enhancement is attributed to the synergy between OWP and alginate. It is noteworthy that the biosorbent synthesis process, particularly in OWPSA, contributes to a reduction in adsorption capacity and the availability of sites for adsorption. This phenomenon likely explains the comparatively lower removal of Cu (II) by OWPSA in contrast to alginate, as the adsorption sites become less accessible. The intricate interplay between the different systems highlights the importance of understanding the synergistic effects of composite adsorbents and underscores the need for a nuanced approach to biosorbent design and synthesis. These findings contribute to the broader understanding of adsorption mechanisms and guide future research in tailoring biosorbents for optimal metal ion removal.

Interestingly, the sorption mechanism identified in our study is characterized by a synergistic interplay of physical and chemical interactions between the adsorbents and Cu^2+^ ions, involving electrostatic attractions, ion exchange, and coordination chemistry. In the case of OWP, sorption is predominantly governed by the functional groups inherent in its components, such as cellulose, hemicellulose, and lignin. These groups, containing hydroxyl and phenolic moieties, engage in interactions with Cu^2+^ ions through a combination of ion exchange and coordination, leading to the formation of complexes on the adsorbent surface. The incorporation of alginate in OWPSA serves to further enhance the sorption mechanism. Alginate, being a polysaccharide with carboxylic groups, introduces additional binding sites for metal ions through ion exchange. The carboxyl groups within alginate have the potential to form complexes with Cu^2+^ ions, thereby contributing to the overall adsorption capacity. Consequently, our sorption mechanism comprises a harmonious combination of ion exchange, coordination, and physical interactions between the functional groups of the adsorbents and Cu^2+^ ions. The inclusion of alginate introduces supplementary ion exchange sites, enriching the overall sorption capacity.

#### 2.3.3. Thermodynamic Parameters

Thermodynamic parameters, including Gibbs free energy ( $\Delta G^{0}$ ), enthalpy ( $\Delta H^{0}$ ), and entropy ( $\Delta S^{0}$ ), were calculated using the following formulas [45,46,47]:

(6) $$\Delta G^{0}=-RTLn\;K_{ads}$$

(7) $$Ln\;K_{ads}=\frac{\Delta S^{0}}{R}-\frac{\Delta H^{0}}{RT}$$

Table 2 gives the thermodynamic parameters of Cu^2+^ adsorption onto OWP and OWPSA, where *K_ads_* means equilibrium adsorption. A negative value for Δ*G*^0^ implies that the adsorption process occurs spontaneously, while a positive value for Δ*H*^0^ suggests that it is endothermic. Furthermore, the correlation between the rise in temperature (*T*) and the negative increase in Δ*S*^0^ provides further evidence that the adsorption process is endothermic. It is worth mentioning that the enthalpy values we obtained: specifically, 2.2384 kJ mol^−1^ for OWP, 15.726 kJ mol^−1^ for OWPSA, and 10.340 kJ mol^−1^ for alginate. The values for OWP and OWPSA fall below the commonly accepted threshold for chemical adsorption (∆*H* > 40 kJ mol^−1^) indicating that the adsorption process between these adsorbents and Cu^2+^ ions is primarily governed by physical interactions [48]. This emphasizes that the adsorbate–adsorbent bonding is indicative of physisorption rather than chemisorption.

### 2.4. Molecular Modeling

In assessing the interactions of alginate, cellulose, hemicellulose, and lignin with Cu^2+^ ions, frontier molecular orbitals (FMOs) were calculated. Key metrics for evaluating the electronic properties of the system, namely the highest-occupied molecular orbital (HOMO) and lowest-unoccupied molecular orbital (LUMO) values, were determined [25,49,50,51]. An increase in the energy required for the electron transition from the ground-state HOMO to the excited-state LUMO signifies improved kinetic stability in the system. The quantized descriptors are presented in Table 3. The analysis of the data reveals that hemicellulose and alginate exhibit the highest HOMO energies, while Cu^2+^ displays the lowest. This observation suggests that Cu^2+^ functions as an effective electron acceptor, and is influenced by the electron-donating properties of hemicellulose and alginate. In other words, the energetic characteristics of these molecular orbitals provide insights into the interactions and electronic properties of the system, helping us to understand the roles of different components in the adsorption process.

For Cu^2+^ and the four chosen compounds, we also determined the HOMO-LUMO gap and the hardness. Lignin, with the lowest energy gap (2.101 eV), is the most reactive molecule, followed by hemicellulose and alginate, with values of 3.677 and 4.403 eV, respectively. As a result, cellulose is regarded as more stable (less reactive) than alginate, hemicellulose, and lignin.

Cu^2+^ is electrophilic, as shown by its low chemical potential (−13.811 eV) and high electrophilicity (23.582 eV). Conversely, the nucleophilic nature of alginate, cellulose, and hemicellulose is supported by their high chemical potential values and low electrophilicity index [52].

**Figure 8:**
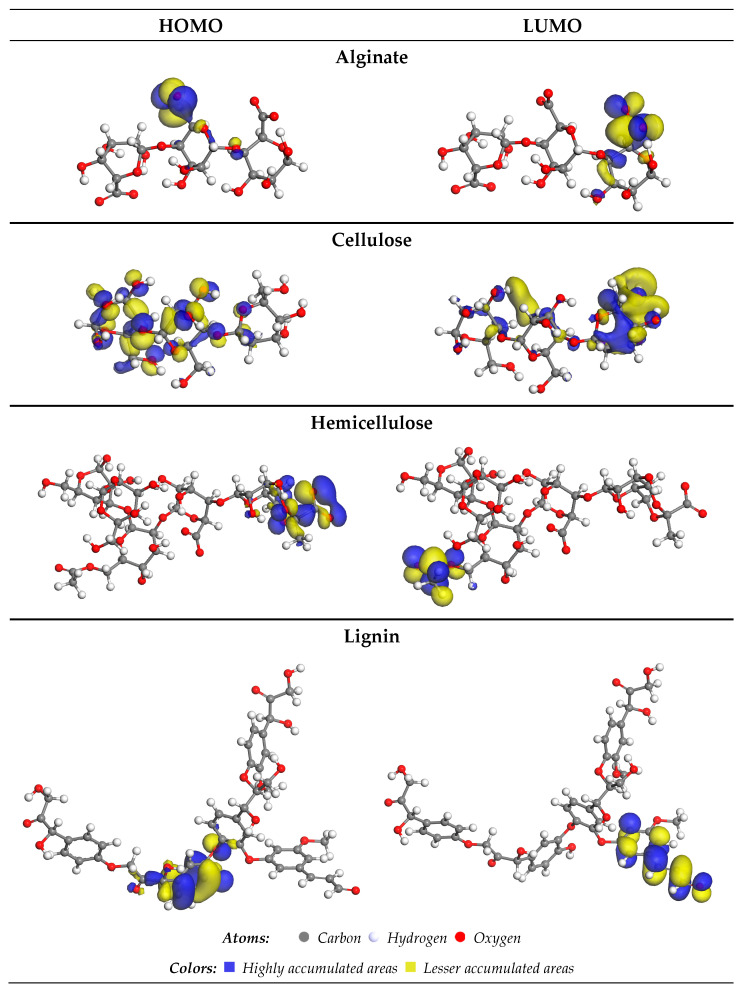
HOMO and LUMO orbitals of lignin, alginate, cellulose, and hemicellulose.

In Figure 8, the highest-occupied molecular orbitals (HOMOs) and lowest-unoccupied molecular orbitals (LUMOs) of alginate, cellulose, hemicellulose, and lignin are presented. These orbitals offer a visual representation of the electronic structure and reactivity of the molecules.

The distinct patterns and energy levels of HOMO and LUMO provide insights into the potential interactions and electron transfer processes between these biomolecules and Cu^2+^ ions. Analyzing these orbitals aids in understanding the electronic properties that govern the reactivity and binding affinity of the molecules with target ions.

The HOMO (+) positions for alginate and hemicellulose are identified on carbon atoms connected to oxygen (O-) and (O=) atoms within the COOH group. In the case of cellulose, the HOMO (+) was located on the carbon coupled with an oxygen atom (OH group). Simultaneously, for lignin, it is situated on the carbon atoms linked to the C=C group.

Positive σ-values of COSMO-RS represent negative polarity and negative values represent positive polarity [53,54,55,56]. The “σ-profile” curves are used to evaluate metal ion interactions and determine the global polarity of molecules [57,58,59,60].

**Figure 9:**
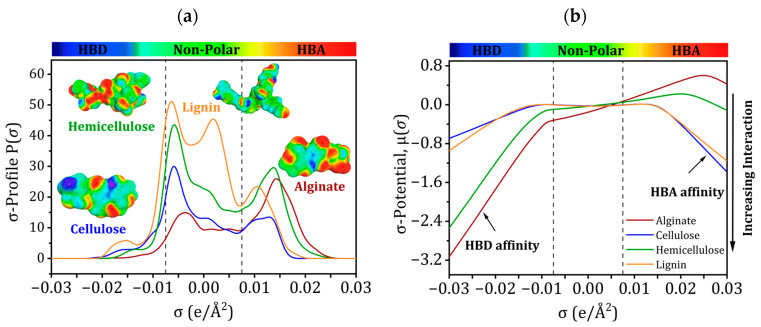
(**a**) Sigma profiles and (**b**) Sigma potentials of alginate, cellulose, hemicellulose, and lignin.

The “σ-profile”, as depicted in Figure 9a, can be categorized into three distinct regions: the hydrogen acceptor area (where σ values range from +0.075 to +0.0300 e/Å^2^), the non-polar region (where σ values fall between −0.0075 and +0.0075 e/Å^2^), and the HBD region (where σ values fall between −0.0300 and −0.0075 e/Å^2^) [61]. The non-polar region has the most prominent peaks for the four molecules, while the hydrogen acceptor region contains the weakest. The HBD zone is devoid of any discernible peaks.

Figure 9b shows how “σ-potential” affinities were used to confirm the “σ-profile” tendencies. Higher positive values indicate more repulsion, whereas higher negative values indicate greater affinity [57]. Weakly negative “σ-potentials” may be seen in non-polar areas (Figure 9b), suggesting lower affinities. In contrast to their strong affinity for HBA, alginate and hemicellulose prefer HBD. In contrast, the frontier molecular orbital research results demonstrate that cellulose and lignin strongly attract HBA and have less affinity for HBD.

**Figure 10:**
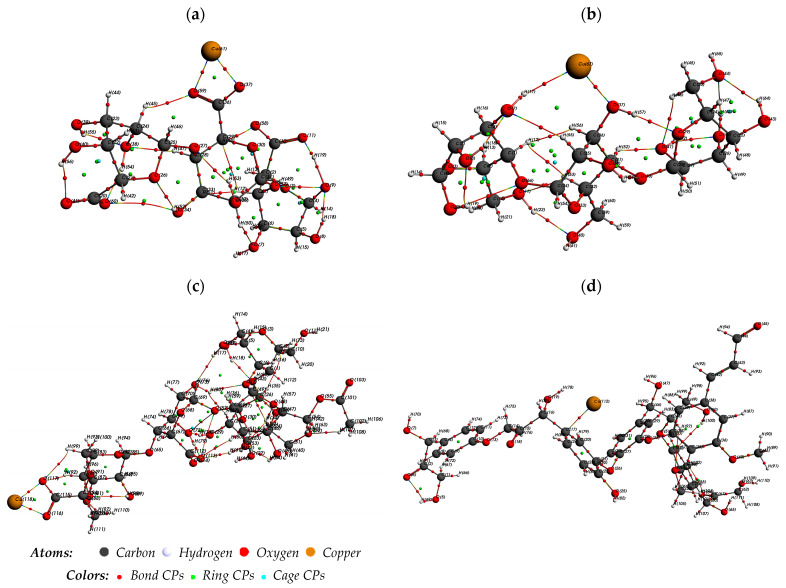
Molecular graph of (**a**) Cu^2+^–alginate, (**b**) Cu^2+^–cellulose, (**c**) Cu^2+^–hemicellulose, and (**d**) Cu^2+^–lignin derived from AIM analysis.

An AIM study was performed to further comprehend the intermolecular interactions within the complexes under investigation. The molecular structure of the enhanced compounds is shown in Figure 10. Table S2 in the Supplementary Material presents the computed values of ρ(rc), ∇^2^ρ(r_c_), and H(r_c_). The observed values of ρ(r_c_) ranged from 1.77 × 10^−3^ to 7.84 × 10^−2^ a.u., indicating they were very minor. Adsorbents and Cu^2+^ interact weakly when ∇^2^ρ(r_c_) is positive.

Especially concerning carbon–Cu bonds, the highest ρ(rc) values were observed at bond-critical points (BCPs), measuring around 10^−2^ atomic units for BCP123 in cellulose and alginate, BCP285 in hemicellulose, and BCP190 in lignin. According to these values, hydrogen bonding interactions are probable. The BCPs had the greatest absolute E HB values within their respective systems: 5.70 × 10^−2^ a.u. for lignin ‘BCP190’, 3.70 × 10^−2^ a.u. for alginate ‘BCP123’, 3.30 × 10^−2^ a.u. for cellulose ‘BCP123’, and 5.50 × 10^−2^ a.u. for hemicellulose ‘BCP285’. These magnitudes indicate that these specific BCPs formed the strongest bonds among all BCPs.

Additionally, the G(rc)/|V(rc)| values for the four mentioned BCPs—cellulose ‘BCP123’, alginate ‘BCP123’, hemicellulose ‘BCP285’, and lignin ‘BCP190’—are close to one for cellulose and less than one for alginate, hemicellulose, and lignin, respectively. This confirms the weak interaction of Cu^2+^ with cellulose ‘BCP123’, while alginate, hemicellulose, and lignin exhibit strong interactions with a partially covalent character. Specifically, in the Cu^2+^ lignin system, the dispersive forces resulting from the kinetic energy G(rc) between Cu (112) and C are less significant than the cohesive forces provided by the potential |V(rc)| (17).

## 3. Materials and Methods

**Figure 11:**
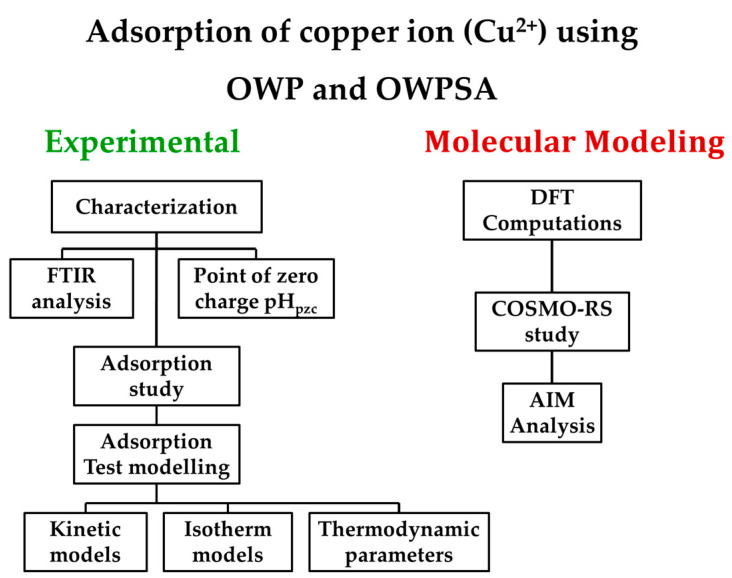
A graphical representation of the research method used for this study.

Figure 11 illustrates the comprehensive methodology employed in this study and elucidates the sequential steps encompassing both the experimental and molecular modeling aspects. The subsequent sections provide a detailed breakdown of each step and elucidate the procedures and methodologies applied in the experimental and molecular modeling phases.

### 3.1. Chemical Reagents for Investigating Copper Ion Adsorption

The sodium alginate (C_6_H_7_O_6_Na), hydrochloric acid (HCl), sodium hydroxide (NaOH), copper (II) chloride (CuCl_2_·2H_2_O), and calcium chloride (CaCl_2_) utilized in this investigation were all of analytical grade and purchased from Sigma Aldrich (St. Louis, MO, USA).

### 3.2. Creation of Adsorbents from Olive Waste

The adsorbent used in this experiment was derived from olive waste collected at a modern oil mill in Beni Ouarthilane, Setif, Algeria. Specifically, the adsorbent was created from discarded olive pulp obtained after the first round of centrifugation, comprising pits, peels, and olive leaves. The process involved a series of hot distilled-water washes, followed by air drying and boiling. After centrifugation at 6000 rpm, the resulting powder was obtained through drying at 353 K, crushing, and sifting. This olive waste served as a base alone (OWP) or combined with sodium alginate (OWPSA) to create two distinct adsorbents.

### 3.3. Biosorbent Bead Synthesis

To prepare the biosorbent beads, the reclaimed powder with a particle size of 0.1 mm was combined with a solution containing 2 g of sodium alginate (SA) in 100 mL of distilled water. The mixture underwent stirring for 1 h until homogeneity was achieved. Following this, the resulting mixture (OWPSA) was mechanically stirred for an additional 24 h after being dispensed from a syringe into a 4% (*m*/*v*) calcium chloride (CaCl_2_) solution. Subsequently, the formed beads were separated through centrifugation, and any residual CaCl_2_ was thoroughly washed away with distilled water. Finally, the beads were air-dried for 48 h at 303 K, resulting in a produced powder with an approximate particle size of 3 mm.

### 3.4. Characterization of Adsorbent Properties

#### 3.4.1. Determining the Point of Zero-Charge pH_pzc_

The pH_pzc_, which signifies the equilibrium between positive and negative charge adsorption on the adsorbent surface, is an essential metric. This parameter is crucial in adsorption methods that rely on electrostatic force. The material has a positive charge if the medium pH is lower than pH_pzc_ and becomes negatively charged if the pH surpasses it [62,63]. In closed flasks containing 50 mL of distilled water, the pH was changed from 1 to 12 by adding HCl or NaOH solutions (0.1 M or 1 M). Following a twenty-four-hour duration at room temperature, continuous agitation was implemented on the solutions within each vial, each containing 50 mg of the adsorbent. At the intersection of the *x*-axis and the ∆pH = f (pHi) graph, the pH_pzc_ value was determined.

#### 3.4.2. Investigating Adsorbent Surface Chemistry

Infrared spectroscopy (IR) analysis using KBr pellets and a Shimadzu FTIR-8400S (Kyoto, Japan) determined the principal chemical functionalities on the adsorbents’ surface.

### 3.5. Experimental Adsorption Study

Firstly, adsorption experiments were conducted at room temperature (303 K) using an initial concentration of *C*_0_ = 50 mg/L copper (II) chloride (CuCl_2_) solution, with the pH varying from 3 to 5. Then, 20 mg of adsorbent was added to 20 mL of the solution and the mixture was continuously swirled at 200 revolutions per minute for 10 h. After testing, samples were centrifuged for 30 min at 6000 rpm to remove the solids, and atomic absorption spectrometry (AAS) was used to analyze the remaining Cu^2+^ amounts at the equilibrium (*C_e_* in mg/L) at 249.22 nm. The adsorption capacity *q_e_* (mg/g) was calculated using Equation (1):

(8) $$q_{e}=\frac{\left(C_{0}-C_{e}\right)V}{m}$$

where *C*_0_ (mg/L) is the initial concentration, *V* (L) is the volume of the solution, and *m* (g) the amount of the adsorbent.

Adsorption kinetic tests were performed by fixing the pH at 5 with the same temperature (303 K), initial concentration (*C*_0_ = 50 mg/L), and solid–liquid ratio (1 g/L) conditions.

The isotherm study was performed at pH = 5 at 293, 303, and 313 K for a contact time of 24 h. The tests were performed on OWP and OWPSA adsorbents, and additional tests were carried out on pure alginate to isolate and quantify its contribution to the composite beads.

### 3.6. Molecular Insights

#### 3.6.1. Quantum Chemical Descriptors and Conductor-like Screening Model for Realistic Solvents Approach

The implementation of theoretical calculations was undertaken to complement the experimental adsorption data. The selection of adsorbent components, namely hemicellulose, lignin, alginate, and cellulose, was based on the inherent composition of vegetal material. The choice aligns with the typical constituents found in olive waste, as documented in previous studies [62,63,64]. Given our laboratory-focused objectives, an in-depth chemical composition analysis of the vegetal material was not pursued.

The def-TZVP basis functions and the geometric optimization of three-dimensional structures [61] at the GGA-BP86 level of theory were accomplished with TmoleX v 4.5.1 software. The interaction between the adsorbent components and copper ions was examined using quantum chemical reactivity descriptor analysis. The analyzed characteristics included *E_HOMO_*, *E_LUMO_*, and $\Delta E_{gap}$ (eV) (denoting the highest-occupied and lowest-unoccupied molecular orbital, and gap energies), chemical potential (*μ*, eV), electronegativity ( χ , eV), global hardness (*η*, eV), the electrophilicity index (*ω*, eV), and the maximum amount of electronic charge index (Δ*N_max_*, eV) [65,66].

(9) $$\Delta E_{gap}=E_{LUMO}-E_{HOMO}$$

(10) $$\mu =-\chi =\frac{E_{LUMO}+E_{HOMO}}{2}$$

(11) $$\eta =\frac{E_{LUMO}-E_{HOMO}}{2}$$

(12) $$\omega =\mu^{2}/2\eta$$

(13) $$\Delta N_{max}=\frac{\chi_{Cu-}\chi_{adsorbent}}{2\left(\eta_{Cu}+\eta_{adsorbent}\right)}$$

**Figure 12:**
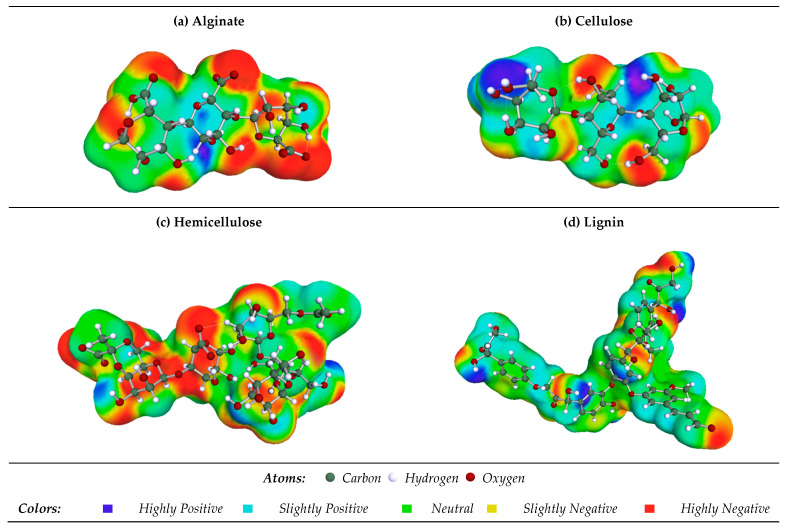
The 3D structures of the modeled molecules.

In solution, COSMO-RS succeeds at computing a wide range of chemical characteristics [67]. It models the chemical potential, sigma charge density, and molecule interactions [27,68]. The BIOVIA COSMOtherm program (version 2022) was used to read the optimized COSMO files. The sigma profiles and potentials for the four investigated compounds were created with the help of this program. Figure 12 shows a schematic depiction of the three-dimensional architectures of these molecules. This COSMO-RS method allows for a thorough investigation of the sigma charge density and potentials for the molecules under consideration, thanks to its extensive knowledge of molecular interactions.

#### 3.6.2. Exploring Molecular Interactions: Atom-in-Molecule (AIM) Theory

The atom-in-molecule (AIM) theory is useful for calculating approximate intermolecular forces. This hypothesis employs each molecule’s electron densities ρ(r; X) to examine molecular bonding interactions. In AIM, the bond-critical point (BCP), where the electron density is at its lowest, is the most important analytical point. When the number is large and the ∇^2^ρ(r) is small, it suggests a strong covalent (polar) bond. A positive ∇^2^ρ(r) at a low value indicates that the kinetic energy G(r) is greater than the potential energy V(r) [69].

The Amsterdam Density Functional (ADF) program (version 2013) [70] was used for the AIM computations. To optimize the molecule structures, the DFT method was used; the def-TZVP basis set was used to study the exchange–correlation impacts of these factors [71]. This method elucidates the type and strength of chemical bonds in the system under study by providing precise knowledge of interactions at the molecular level.

## 4. Conclusions

This research study used olive waste powder (OWP) and sodium alginate to produce two adsorbents (OWPSA). On these substances, adsorption tests involving Cu^2+^ ions in synthetic wastewater were subsequently conducted. A comprehensive investigation was undertaken to examine the molecular interactions between Cu^2+^ ions and OWP and OWPSA adsorbents via molecular modeling. In the instance of OWPSA, the solid substances were represented by combinations of cellulose, hemicellulose, lignin, and alginate. We used statistical physics, density functional theory (DFT) calculations, atom-in-molecule (AIM) analysis, and the conductor-like screening model for real solvents (COSMO-RS) for our investigation.

The combined experimental and modeling outcomes led to the following noteworthy conclusions.

Cu^2+^ removal efficiency reached approximately 79.54% for OWP and 94.54% for OWPSA. The presence of alginate in OWPSA contributed to increased adsorption capacity, albeit constrained by the bead production process.

Cu^2+^ adsorption capacity exhibited a pH-dependent increase, likely attributed to reduced competition from H^+^ ions, and a temperature-dependent rise, possibly due to increased active adsorption sites.

The adsorption kinetics revealed comparable initial behaviors for OWP and OWPSA, but OWPSA exhibited a slower approach to saturation. The application of the pseudo-second-order (PSO) model provided a meaningful interpretation of the kinetics, demonstrating its effectiveness in describing the dynamic copper adsorption behavior of both OWP and OWPSA. The observed slower kinetics of OWPSA suggest that the rate-limiting step in the adsorption process is likely associated with chemisorption or a more intricate mechanism, contributing to a delayed attainment of saturation compared to OWP.

Statistical physics isotherm models, specifically the single-layer model coupled to real gas (SLMRG), demonstrated a robust fit to the experimental data, thereby offering valuable insights into the underlying phenomenology. The SLMRG isotherms emerged as the most accurate representations of the adsorption equilibria, revealing the maximum adsorption capacities for OWP and OWPSA, respectively.

Thermodynamic parameters confirmed the spontaneity and endothermic nature of the processes governed by physical interactions.

DFT calculations, COSMO-RS study, and AIM analysis collectively affirmed the substantial interaction between alginate, cellulose, hemicellulose, lignin, and Cu^2+^, establishing their physically interactive nature.

AIM analysis conclusively confirmed the robust interaction of Cu^2+^ between alginate, hemicellulose, and lignin affirming their inherent physical interactivity. Additionally, it validated the comparatively weaker interaction of Cu^2+^ with cellulose.

In conclusion, this study aimed to explore the synergistic application of heavy metal adsorption from wastewater through a combination of experimental tests and theoretical calculations. While both experimental and theoretical approaches were employed, it is acknowledged that the synergistic application and the conclusive demonstration of their combined effectiveness may require further refinement and investigation. The results obtained provide valuable insights, but the full realization of a synergistic application could benefit from additional research and methodological enhancements.

## Figures and Tables

**Table 1:** SLMRG fitting parameters of Cu^2+^ isotherms for OWP, OWPSA and alginate.

$T\;\left(K\right)$	OWP			OWPSA			Alginate		
	293 K	303 K	313 K	293 K	303 K	313 K	293 K	303 K	313 K
n	2.037	2.422	1.787	2.860	2.874	2.0020	0.696	0.614	0.481
$N_{m}\;\left(\mathrm{mg}/g\right)$	50.391	45.050	73.937	44.712	48.622	67.544	314.370	424.471	532.179
*Q_sat_* (mg/g)	102.647	109.111	132.125	127.869	139.755	135.224	218.707	260.497	256.085
$a\;\left(J\cdot L/\mathrm{mg}\right)$ ⨯ 10^−23^	8.46	8.47	8.48	8.75	8.60	9.60	10.59	54.90	67.10
$b\;\left(L/\mathrm{mg}\right)$	0.0080	0.0080	0.0041	0.0071	0.0077	0.0096	0.0084	0.0084	0.0087
$w\;\left(\mathrm{mg}/L\right)$	9.283	11.646	14.424	10.788	12.726	9.032	70.965	12.481	38.338
R^2^	0.9756	0.9910	0.9935	0.9960	0.9869	0.9860	0.9787	0.9733	0.9873
RMSE	4.7368	3.2981	2.6754	3.5125	10.3826	6.8785	12.8099	15.1116	9.7747

**Table 2:** Thermodynamic parameters for Cu^2+^ adsorption onto OWP, OWPSA, and Alginate.

Adsorbent	T [K]	Kads	ln Kads	ΔG° [kJ/mol]	ΔH° [kJ/mol]	ΔS° [kJ/mol·K]
OWP	293	1.4981	0.4042	−0.985	2.2384	0.011
OWP	303	1.5442	0.4345	−1.095	2.2384	0.011
OWP	313	1.5887	0.4629	−1.205	2.2384	0.011
OWPSA	293	1.6829	0.5205	−1.268	15.726	0.058
OWPSA	303	2.0825	0.7336	−1.848	15.726	0.058
OWPSA	313	2.5422	0.933	−2.428	15.726	0.058
Alginate	293	1.2283	0.2057	−0.501	10.34	0.037
Alginate	303	1.4131	0.3458	−0.871	10.34	0.037
Alginate	313	1.6111	0.4769	−1.241	10.34	0.037

**Table 3:** Calculated quantum parameters and electronic properties of Cu^2+^, alginate, cellulose, hemicellulose, and lignin.

	$E_{HOMO}$	$E_{LUMO}$	$\Delta E_{gap}$	μ	χ	η	ω	$\Delta N_{max}$
Cu^2+^	−17.855	−9.767	8.088	−13.811	13.811	4.044	23.582	3.415
Alginate	−4.928	−0.525	4.403	−2.727	2.727	2.201	1.689	1.239
Cellulose	−5.776	0.260	6.036	−2.758	2.758	3.018	1.260	0.914
Hemicellulose	−4.626	−0.950	3.677	−2.788	2.788	1.838	2.114	1.517
Lignin	−5.211	−3.110	2.101	−4.160	4.160	1.050	8.239	3.960

## Data Availability

Data are contained within the article and supplementary materials.

## Supplementary Materials

The following supporting information can be downloaded at: https://www.mdpi.com/article/10.3390/ijms25021028/s1.
