# Correction: Elboughdiri et al. Comprehensive Investigation of Cu^2+^ Adsorption from Wastewater Using Olive-Waste-Derived Adsorbents: Experimental and Molecular Insights. *Int. J. Mol. Sci.* 2024, *25*, 1028

**DOI:** 10.3390/ijms262210933

**Published:** 2025-11-12

**Authors:** Noureddine Elboughdiri, Hana Ferkous, Karima Rouibah, Abir Boublia, Amel Delimi, Krishna Kumar Yadav, Alessandro Erto, Djamel Ghernaout, Alsamani A. M. Salih, Mhamed Benaissa, Yacine Benguerba

**Affiliations:** 1 Chemical Engineering Department, College of Engineering, University of Ha’il, P.O. Box 2440, Ha’il 81441, Saudi Arabiasamani15@hotmail.com (A.A.M.S.); m.benaissa@uoh.edu.sa (M.B.); benguerbayacine@yahoo.fr (Y.B.); 2 Laboratoire de Génie Mécanique et Matériaux, Faculté de Technologie, Université de Skikda, Skikda 21000, Algeria; h.ferkous@univ-skikda.dz (H.F.); a_delimi03@yahoo.fr (A.D.); 3 Laboratory of Materials-Elaborations-Properties-Applications (LMEPA), University of MSBY Jijel, PB98 Ouled Aissa, Jijel 18000, Algeria; karima.rouibah@univ-jijel.dz; 4 Laboratoire de Physico-Chimie des Hauts Polymères (LPCHP), Département de Génie des Procédés, Faculté de Technologie, Université Ferhat ABBAS Sétif-1, Sétif 19000, Algeria; abir.boublia@univ-setif.dz; 5 Faculty of Science and Technology, Madhyanchal Professional University, Ratibad, Bhopal 462044, India; envirokrishna@gmail.com; 6 Environmental and Atmospheric Sciences Research Group, Scientific Research Center, Al-Ayen University, Thi-Qar, Nasiriyah 64001, Iraq; 7 Dipartimento di Ingegneria Chimica, dei Materiali e della Produzione Industriale, Università di Napoli Federico II, 80125 Napoli, Italy; 8 Laboratoire de Biopharmacie et Pharmacotechnie (LBPT), Université Ferhat ABBAS Sétif-1, Sétif 19000, Algeria

In the original publication [1], there was a mistake in the legend for Table 2. The authors mistakenly reported erroneous values of ΔG°. This has now been corrected. We recalculated all thermodynamic parameters based on Equation (7) and updated Table 2 accordingly. The correct legend appears below. The authors state that the scientific conclusions are unaffected. This correction was approved by the Academic Editor. The original publication has also been updated.

## Figures and Tables

**Table 2:** Thermodynamic parameters for Cu^2+^ adsorption onto OWP, OWPSA, and Alginate.

Adsorbent	T [K]	Kads	ln Kads	ΔG° [kJ/mol]	ΔH° [kJ/mol]	ΔS° [kJ/mol·K]
OWP	293	1.4981	0.4042	−0.985	2.2384	0.011
OWP	303	1.5442	0.4345	−1.095	2.2384	0.011
OWP	313	1.5887	0.4629	−1.205	2.2384	0.011
OWPSA	293	1.6829	0.5205	−1.268	15.726	0.058
OWPSA	303	2.0825	0.7336	−1.848	15.726	0.058
OWPSA	313	2.5422	0.933	−2.428	15.726	0.058
Alginate	293	1.2283	0.2057	−0.501	10.34	0.037
Alginate	303	1.4131	0.3458	−0.871	10.34	0.037
Alginate	313	1.6111	0.4769	−1.241	10.34	0.037
