# Supplementary material for: Comprehensive Investigation of Cu2+ Adsorption from Wastewater Using Olive-Waste-Derived Adsorbents: Experimental and Molecular Insights
Source: Int J Mol Sci. 2024 Jan 14;25(2):1028. doi: 10.3390/ijms25021028 (PMC10816160; doi:10.3390/ijms25021028)
Supplement: Supplementary file 1 [file ijms-25-01028-s001.zip › ijms-2753042-supplementary.pdf]

Supplementary Material

Comprehensive Investigation of Cu^{2+} Adsorption from Wastewater Using Olive Waste-Derived Adsorbents: Experimental and Molecular Insights

Noureddine Elboughdiri [1,*], Hana Ferkous [2], Karima Rouibah [3], Abir Boublia [4], Amel Delimi [2], Krishna Kumar Yadav [5,6], Alessandro Erto [7], Djamel Ghernaout [1], Alsamani A. M. Salih [1], Mhamed Benaissa [1] and Yacine Benguerba [1,8]

1 Chemical Engineering Department, College of Engineering, University of Ha'il, P.O. Box 2440, Ha'il 81441, Saudi Arabia; djamel_andalus@yahoo.fr (D.G.); samani15@hotmail.com (A.A.M.S.); m.benaissa@uoh.edu.sa (M.B.); benguerbayacine@yahoo.fr (Y.B.)
2 Laboratoire de Génie Mécanique et Matériaux, Faculté de Technologie, Université de Skikda, Skikda 21000, Algeria; h.ferkous@univ-skikda.dz (H.F.); a_delimi03@yahoo.fr (A.D.)
3 Laboratory of Materials-Elaborations-Properties-Applications (LMEPA), University of MSBY Jijel, PB98 Ouled Aissa, Jijel 18000, Algeria; karima.rouibah@univ-jijel.dz
4 Laboratoire de Physico-Chimie des Hauts Polymères (LPCHP), Département de Génie des Procédés, Faculté de Technologie, Université Ferhat ABBAS Sétif-1, Sétif 19000, Algeria; abir.boublia@univ-setif.dz
5 Faculty of Science and Technology, Madhyanchal Professional University, Ratibad, Bhopal 462044, India; envirokrishna@gmail.com
6 Environmental and Atmospheric Sciences Research Group, Scientific Research Center, Al-Ayen University, Thi-Qar, Nasiriyah 64001, Iraq
7 Dipartimento di Ingegneria Chimica, dei Materiali edella Produzione Industriale, Università di Napoli Federico II, 80125 Napoli, Italy; aleserto@unina.it
8 Laboratoire de Biopharmacie et Pharmacotechnie (LBPT), Université Ferhat ABBAS Sétif-1, Sétif 19000, Algeria
* Correspondence: ghilaninouri@yahoo.fr; Tel.: +966-549-571015

Table S1. The kinetic parameters of PS1 and PS2 for OWP and OWPSA.

Adsorbents	Experimental			PS1		
	$Q_{e,\ exp\ (mg/g)}$			k_1(min^{-1})	$Q_{e,\ PS1\ (mg/g)}$	R^2
OWP	39.77			0.0139	44.99	0.8873
OWPSA	47.27			0.00575	33.28	0.9840
	**PS2**			**Intraparticle diffusion model**		
	k_2 ($g.mg^{-1}.\ min^{-1}$)	$Q_{e,\ PS2\ (mg/g)}$	R^2	K_{id} ($mg.g^{-1}.min^{-0.5}$)	C	R^2
OWP	0.0005	43.12	0.9935	1.897	4.910	0.94936
OWPSA	0.0004	49.58	0.9896	2.082	6.629	0.96055

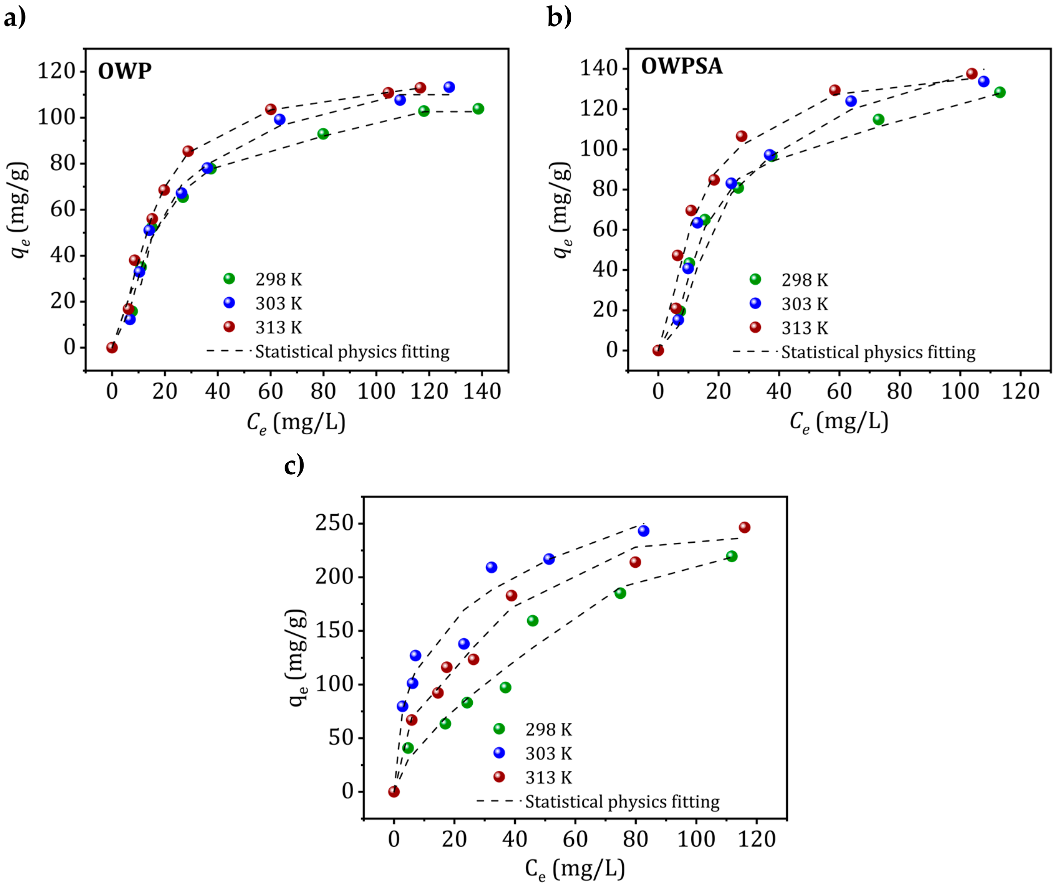

Figure S1. Adsorption isotherms modeling of Cu^{2+} isotherms onto a) OWP, b) OWPSA, and c) alginate adsorbents at various temperatures.

Table S2. Topological parameters at BCP of interaction contacts in considered complexes.

BCP	$\rho(r_c)$	$\nabla^2\rho\ (r_c)$	$G(r_c)$	$V(r_c)$	E_{HB}	$H(rc) = G(rc) + V(rc)$	$G(rc)/\lvert V(rc)\rvert$
**Cu^{+2} −alginate**							
123	0.0784	0.3740	0.1040	-0.1140	-0.0570	-0.0100	0.91
124	0.0776	0.3720	0.1030	-0.1120	-0.0560	-0.0090	0.92
**Cu^{+2} −cellulose**							
98	0.0177	0.0240	0.0080	-0.0090	-0.0050	-0.0010	0.84
123	0.0541	0.2640	0.0660	-0.0670	-0.0330	-0.0002	1.00
**Cu^{+2} −hemicellulose**							
285	0.0762	0.3660	0.1000	-0.1090	-0.0550	-0.0090	0.92
286	0.0720	0.3420	0.0930	-0.1000	-0.0500	-0.0070	0.93
**Cu^{+2} −lignin**							
190	0.0659	0.1460	0.0550	-0.0740	-0.0370	-0.0190	0.75
